# Sexual Violence and Associated Factors Among Female Students at Debre Berhan University, Ethiopia

**DOI:** 10.7759/cureus.16189

**Published:** 2021-07-05

**Authors:** Semira Mehammed Hassen, Bedru Hussen Mohammed

**Affiliations:** 1 Obstetrics and Gynecology, School of Nursing, The University of Hong Kong, Hong Kong, HKG; 2 Epidemiology and Public Health, School of Public Health, The University of Hong Kong, Hong Kong, HKG

**Keywords:** sexual violence, female students, debre berhan university, ethiopia

## Abstract

Background and objective

Sexual violence is a common and serious public health issue affecting millions of women at all stages of their lives. Studies have shown that women in Ethiopia are among the highly affected. There is no study in the literature as yet on the prevalence and factors associated with sexual violence among female students of Debre Berhan University (DBU), Ethiopia. In light of this, this study aimed to assess the prevalence of sexual violence and associated factors among female students at DBU.

Methods

A Cross-sectional institution-based study was conducted in May 2016 among female regular undergraduate students of DBU. Data was collected using a self-administered questionnaire. Descriptive, binary, and multivariable logistic regression analyses were carried out.

Results

A total of 627 female students completed the questionnaire (response rate: 91.5%). The mean (SD) age of the students was 20.7 (1.65) years. More than half (54.9%) of the students have been victims of sexual violence in their life. More than one-third (35.6%) of the students have experienced rape, attempted rape, or sexual harassment while they are in DBU. Sexual harassment was the most common form of sexual violence ever experienced by female students (51.8%) followed by attempted rape (12.8%) and rape (9.8%). More than half of the rape victims (35, 57.3%) did not share their experiences with anyone. Multiple logistic regression analyses revealed that Khat-chewing habit and marital status were significantly associated with rape.

Conclusion

Sexual violence, in general, is high among female students at DBU. Most of the sexual violence occurs outside of the campus and the perpetrators are mostly known to the victims and are trusted and loved by them. Further broad and longitudinal studies are needed to determine the predictors of the problem among female students at DBU and Ethiopia as a whole.

## Introduction

Violence against women and girls is a global issue and one of the most prevalent human rights violations; it entails denying women their right to enjoy fundamental freedom, dignity, equality, self-worth, and security. Women at any stage in their lives can be affected and it can occur in various forms, which may involve physical, psychological, sexual, social, and economic abuse. Violence against women has a huge impact on every aspect of women’s lives, from their personal health, their psychological health, and self-worth to the safety of their families, and even their ability to earn a living. It is also associated with an increased risk of sexual and reproductive health issues, with both immediate and long-term consequences [1].

Sexual violence is the most widespread form of violence against women [1]. The World Health Organization (WHO) has defined it as any sexual act, attempt to obtain a sexual act, unwanted sexual comments or advances, or acts to traffic, or otherwise directed, against a person’s sexuality using force, by any person regardless of their relationship to the victim, in any setting, including but not limited to home and work [1]. It is the most persistent yet underestimated social and health problem that occurs in pandemic proportions [2], and various studies have shown that women less than 25 years of age are the most common victims of sexual violence [1,2].

The WHO multi-country study on women’s health and domestic violence against women in 2004 documented the result of data from 10 countries (15 sites), including Ethiopia. The high rates of gender-based violence documented by the study (as high as 62%) experienced by girls and women were of great concern [3]. The prevalence of sexual violence is generally high in Africa and ranges from 16% in Cameroun, 23% in Sierra Leone, 34.4% in Ethiopia, and 49% in Ghana to 65.6% in Zimbabwe and 67% in Botswana [2-9].

Studies indicate that schools and universities are places where sexual violence rampantly and routinely occurs [1,2]. In Ethiopia, many studies have indicated a high prevalence of gender-based violence in general and sexual violence in particular at educational institutions. A 2008 national study conducted among a large sample of students reported that about a quarter of schoolgirls have been sexually attacked by either students or teachers [10]. In a cross-sectional survey conducted among female students at the Addis Ababa University in 2004, the lifetime prevalence of completed and attempted rape was 12.7% and 27.5%, respectively, whereas 58% of the students reported experiencing lifetime sexual harassment in some form [11]. A similar study conducted among female students of Haromaya University showed that 3% of females experienced rape in their lifetime and 27.8% of students experienced uninvited sexual overtures such as verbal jokes and direct solicitation for sexual intercourse, and 19.3% encountered unwelcome touches on their bodies [12].

Mostly, sexual violence is rarely reported worldwide [13]. Victims usually prefer to keep their painful experiences to themselves. The reasons for this could be indecision, inability to verbalize their problems in the open, fear of the attacker, or the fear that they could be asked to appear as a witness in a trial to validate their accounts. Data on sexual violence are usually gathered from clinical settings, the police, surveys, and nongovernmental organizations. Therefore, the global magnitude of the problem is thought to be higher than reported, and hence sexual violence has been called a "silent epidemic" [13-15].

Though they are not fully investigated, several of the available studies show that there is no single factor to fully account for violence perpetrated against women. There are several factors that increase the risk of someone being coerced into sex. Generally, violence against women is the result of a complex interplay of individual, relationship-related, social, cultural, and environmental factors [1,16,17].

Studies have shown that sexual violence against women leads to a number of problems including unwanted pregnancy, increased risk of HIV/AIDS infection and other sexually transmitted infections (STIs), gynecological problems, feeling of worthlessness, depression, fear and guilty feeling about sex, powerlessness, shame, difficulty in trusting people, post-traumatic stress disorder (PTSD), and even suicide [5,7,16-20]. On top of that, it also exposes girls and women in schools to social consequences ranging from poor educational achievement to social withdrawal, having multiple partners, rejecting friends, drug and alcohol abuse, and even prostitution [15,17,21].

Scarce literature is available on the nature and extent of sexual violence against female students in colleges and universities in Ethiopia. Even though previous studies indicate that schools and universities are highly susceptible to sexual and gender-based violence, the problem has not been adequately addressed in the educational sector [3-5,7,8,10-12,19,22-25]. Female students in higher institutions are vulnerable to sexual violence due to various reasons in Ethiopia. High school graduates usually have to travel far away from their home and family for the first time in their lives. Gathering data concerning sexual violence among female students is vital in determining the magnitude of sexual violence and the factors associated with it. To the best of our knowledge, studies related to sexual violence have not been conducted at Debre Berhan University (DBU). This lack of reliable data on the prevalence of sexual violence and associated factors among female students at DBU drove us to undertake this study. The study provides insights that would be helpful in designing and implementing evidence-based interventions at the institutional level, and regional as well as national level programs to create a conducive environment for female students, especially in teaching-learning institutional settings.

## Materials and methods

Study design and setting

An institution-based cross-sectional study was conducted in May 2016 to assess the level of sexual violence and identify the associated factors among female students registered at DBU located in the Amhara Region, North Shoa Zone, 130 km North of Addis Ababa, Ethiopia.

Sample size and sampling technique

The sample size for the study was determined by using the single population proportion formula [26], based on the 41.8% prevalence of sexual violence from a study conducted among female students at the Addis Ababa University [11]. A 5% margin of error and 95% level of confidence were assumed. Based on these assumptions, the calculated sample size was 374. As per the sampling technique, we used a correction formula, and a 10% of non-response rate was added, and a design effect of 1.5 was used, which gave us a final sample size of 570 female students.

The study used a multistage sampling technique. The number of female students registered full-time in the undergraduate programs for the 2015/16 academic year in each department was obtained from the registrar and stratified based on year of study from the first up to the fifth year. After proportional allocation to each year, a cluster sampling method was employed where each department was considered as a cluster. A simple random sampling technique was used for the selection of departments; after that, all female students in the randomly selected departments were included as study subjects.

Study variables and operational definitions

Sexual violence (rape, attempted rape, and sexual harassment) was the dependent variable of the study. The independent variables of the study were sociodemographic characteristics (age, residence, religion, relationship status), substance use (cigarette, alcohol, Khat, drugs), family background (income, educational status), year of study, grade, and current living situation. The questions were divided into four sections: (i) sociodemographic characteristics of participants, (ii) substance use among participants, (iii) reproductive history of the participants, and (iv) sexual violence experience of participants. The study used the following operational definition of terms:

Rape: an act of non-consensual sexual intercourse. This can include the invasion of any part of the body with a sexual organ and/or the invasion of the genital or anal opening with any object or body part.

Attempted rape: efforts to rape someone that do not result in penetration.

Sexual harassment: unwanted sexual behavior such as physical contact or verbal comments, jokes, questions, kissing, hugging, and suggestions of sexual nature.

Sexual violence: acts of rape, attempted rape, and/or sexual harassment.

Data collection

The data was collected using a structured, pre-tested self-administered questionnaire adapted from the WHO multi-country questionnaire on violence against women [1].

Sensitive questions such as rape experience were placed later in the questionnaire in order to reduce discomfort and minimize the non-response rate. The questionnaire was translated into Amharic language and back-translated into English to check its consistency and accuracy. The questionnaire was pretested on 23 female students. The result of the pretest was discussed, and relevant changes and corrections were made in the questionnaire as necessary. Five graduating-class midwifery students carried out the data collection with close follow-up by supervisors.

The questionnaire was distributed after gathering study participants in lecture rooms or dormitories. Immediately after the distribution of the questionnaire, an orientation session was conducted to help the students understand the questions well and fill in the questionnaire, and students were asked not to write their names anywhere on the questionnaire. Finally, the students dropped their completed questionnaires into a sealed box placed at the entrance as they left. Students who had no classes during data collection filled in the questionnaire in their dormitories. The questionnaires were later checked for completeness by the investigators.

Statistical analysis

Investigators entered the data into a Microsoft Excel spreadsheet. After the entry, sprucing up and editing were carried out. It was then exported into the IBM SPSS Statistics software version 24 (IBM, Armonk, NY) and analyzed. Descriptive statistics such as means for continuous and proportions for categorical variables including cross-tabulations were used for data summarization. Differences in proportions were compared for significance using the Chi-square test, with a significance level set at p<0.05. When the assumptions of the Chi-square test were not fulfilled, we used Fisher's exact test.

Finally, binary and multivariate logistic regression analyses were used to identify factors associated with sexual violence by controlling for the effect of potential confounding variables. Only those variables with a p-value of less than 0.05 were included in the multivariate logistic regression analysis. Crude and adjusted odds ratio with 95% confidence intervals were used to observe the relative effect of independent variables against the dependent variable. Variables having a p-value of less than 0.05 were considered as predictors of sexual violence.

## Results

Among a total of 685 students approached, 627 female students participated in the study and filled in the questionnaire (response rate: 91.5%). Of the total 627 students, 28.5% (n=179) were in their first year, 32.1% (n=201) in the second year, 18.0% (n=113) in the third year, 8.6% (n=54) in the fourth year, and 12.8% (n=80) were in the fifth year. The mean age of the participants was 20.7 years (SD=1.65; minimum: 18, maximum: 29). Regarding religion, most of the participants (84.8%, n=532) were Orthodox Christians, followed by Protestants and Muslims: 8.0% (n=50) and 6.7% (n=42) respectively. Majority of the students were single (61.7%, n=387), while 34.0% (n=213) were in a non-marital relationship and 3.7% (n=23) were married.

More than half (53%, n=332) of the participants’ place of childhood residence was urban, and that of 47% (n=295) was rural. The majority of the students (55.3%, n=347) currently lived on campus and were non-cafe (did not use the campus mass food), followed by those living on campus and using the cafe (43.5%, n=273), and those living outside the campus (1.11%, n=7). Among the total participants, 118 (18.8%), 13 (2.1%), 15 (2.4%), and 14 (2.2%) reported that they drank alcohol, smoked cigarettes, chewed Khat, and used drugs, respectively.

The majority of the respondents had illiterate parents: fathers 33.7% (n=211) and mothers 41.5% (n=260); followed by those with primary school and college graduate education for fathers (25.2%, n=158), and only primary school for mothers (23.9%, n=150) (Table 1).

**Table 1:** Sociodemographic characteristics of female students of DBU, May 2016 DBU: Debre Berhan University; GPA: grade point average

Variable	Variable category	N (%)
Age in years	18-20	335 (53.4)
	21-29	292 (46.6)
Relationship status	Single	387 (61.7)
	In a relationship but not married	213 (34.0)
	Married	23 (3.7)
	Previously married	4 (0.6)
Religion	Orthodox Christian	532 (84.8)
	Muslim	42 (6.7)
	Protestant Christian	50 (8.0)
	Other	3 (0.5)
Year of study	One	179 (28.5)
	Two	201 (32.1)
	Three	113 (18.0)
	Four	54 (8.6)
	Five	80 (12.8)
Cumulative GPA	≤2.80	256 (48.8)
	>2.80	269 (51.2)
Place of childhood residence	Rural area	295 (47.0)
	Urban area	332 (53.0)
Current living condition	On campus and using café	273 (43.5)
	On campus but non-cafe	347 (55.3)
	Outside campus	7 (1.2)
Parents’ monthly income	≤3,000 Birr	145 (50.2)
	>3,000 Birr	144 (49.8)
Father’s educational status	Illiterate	211 (33.7)
	Primary school	158 (25.2)
	High school	100 (15.9)
	College graduate	158 (25.2)
Mother’s educational status	Illiterate	260 (41.5)
	Primary school	150 (23.9)
	High school	115 (18.3)
	College graduate	102 (16.3)
Cigarette smoking	No	614 (97.9)
	Yes	13 (2.1)
Alcohol consumption	No	509 (81.2)
	Yes	118 (18.8)
Chewing Khat	No	612 (97.6)
	Yes	15 (2.4)
Using drugs (cocaine, shisha, or marijuana)	No	613 (97.8)
	Yes	14 (2.2)
Total		627 (100)

Sexual experience

Only 128 (20.4%) of the students reported having sexual intercourse previously. The mean age of female students at their first sexual intercourse had been 18.1 years (SD=2.92; minimum: seven years, maximum: 24 years). Of those who had already had sexual intercourse, 15 (11.8%) had three or more sexual partners. The most common reason for starting sexual intercourse was personal desire (36, 28.1%); followed by the partner’s promising words (35, 27.3%), marriage (30, 23.4%), and use of force by the perpetrator (13, 10.2%) (Table 2).

**Table 2:** Sexual experience among female students of DBU, May 2016 DBU: Debre Berhan University

Variable	Variable category	N (%)
Ever had sexual intercourse		
	Yes	128 (20.4)
	No	499 (79.6)
Number of sexual partners		
	One	93 (72.7)
	Two	20 (16.6)
	Three	7 (5.5)
	Four or more	8 (6.2)
Reason for starting sexual intercourse		
	Marriage	30 (23.4)
	Personal desire	36 (28.1)
	Promising words from partner	35 (27.3)
	Forced	13 (10.2)
	Peer pressure	10 (7.8)
	Other	4 (3.2)
Total		627 (100)

Sexual violence experience

More than half (54.9%, n=345) of the students had been a victim of at least one of the three sexual violence types in their life. More than one in three (35.6%, n=224) of the students had been victims of rape, attempted rape, or sexual harassment while they were in DBU. Out of the total 627, 61 (9.8%) and 10 (1.6%) students had been victims of completed rape in their life and in DBU respectively. More than one in four of the victims of completed rape had experienced it more than once.

**Figure 1:**
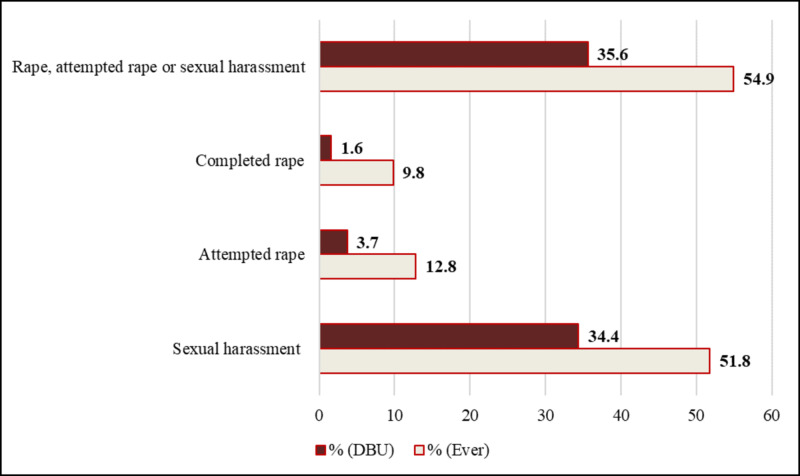
The proportion of different types of sexual violence experience ever and in DBU among female students of DBU, May 2016 DBU: Debre Berhan University

Sexual harassment was the most common type of sexual violence ever encountered by female students, as reported by 51.8% (n=325) of participants, followed by attempted rape (12.8%, n=80), and rape (9.8%, n=61). Most of the victims of rape had been raped once (7.2%), twice (1.8%), and three or more times (0.8%). Of the students who had experienced sexual intercourse, 47.6% (n=61) had been raped at least once in their lifetime (Figure 1). In addition, 13% (n=82) of students reported knowing a female student who had been raped in DBU.

**Figure 2:**
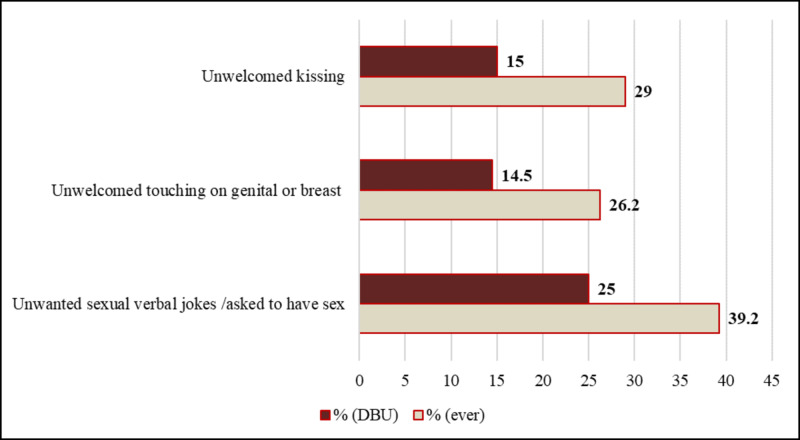
The proportion of different types of sexual harassment experienced ever and in DBU among female students of DBU, May 2016 DBU: Debre Berhan University

More than one in three female students (34.4%) had been a victim of sexual harassment while they were in DBU. Unwanted verbal jokes of sexual nature or requests to have sex were the most common types of sexual harassment experienced by the participants ever (39.2%) and in DBU (25%) (Figure 2).

**Figure 3:**
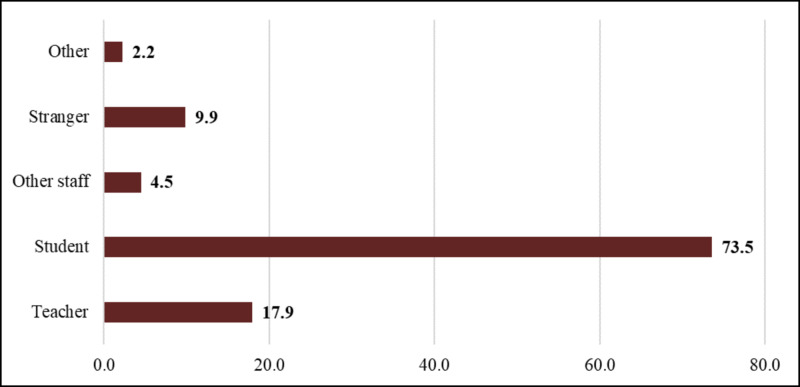
Perpetrators of sexual violence in DBU among female students of DBU, May 2016 DBU: Debre Berhan University

**Figure 4:**
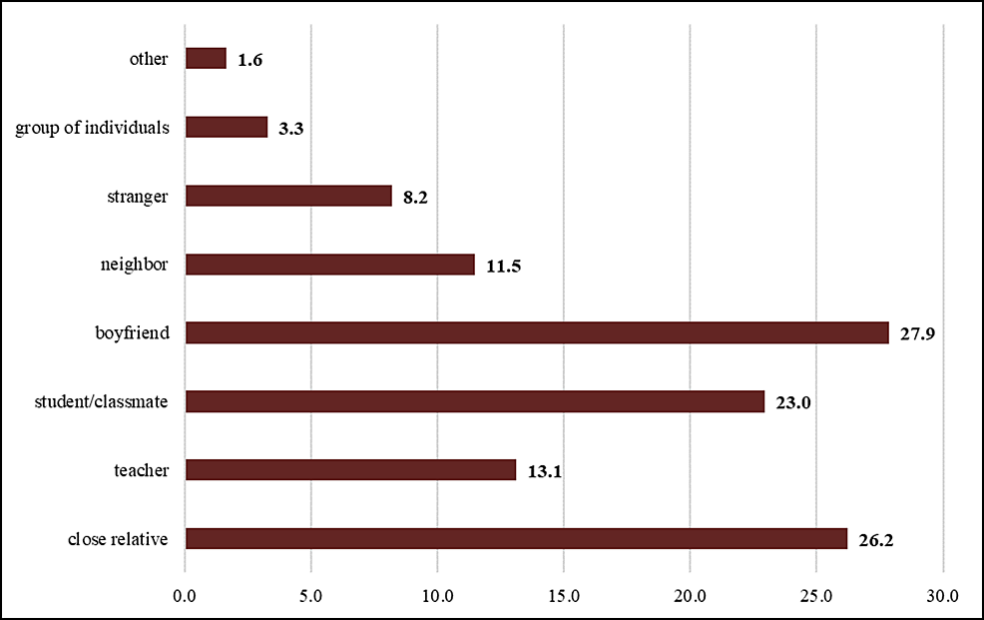
Lifetime perpetrators of sexual violence among female regular students of DBU, May 2016* *Multiple responses were possible for one query, and hence the cumulative percentage may exceed 100% DBU: Debre Berhan University

Fellow students were the most common (73.5%) perpetrators of sexual violence in DBU against female students, followed by teachers (17.9%) (Figure 3). However, boyfriends (27.9%) and close relatives (26.2%) were the most common perpetrators of sexual violence in students’ lifetimes (Figure 4).

Similarly, the mechanisms used to force the victims into sex are different. More than a quarter of victims were forced by hitting or biting; 23.6% (n=13) were violated by making them drunk or intoxicated; and 10.9% (n=6) were lured into sexual acts by promising them to award more marks or grades. The majority of the perpetrators (65.4%, n=46) were older than the victims. Most of the completed rapes (36.1%, n=22) happened in the victims’ house, while 19.7% (n=12) took place in the perpetrators’ house, 18% (n=11) in hotels, 16.4% (n=10) in schools, 13.1% (n=8) in friends’ houses, and 4.9% (n=3) in the university campus.

Reporting of sexual violence

More than half of the victims of sexual violence (57.3%, n=35) did not share the experience with anyone. However, 27.8% (n=17) of the victims shared their situation with friends and 13.1% (n=8) shared it with their families. Most of the victims (98.3%, n=60) did not report to the legal authorities. Out of those who did not share with anyone or to the legal bodies, the main reasons cited were that they did not know what to do (45.7%, n=16), fear of shame (40%, n=14), fear of parents' response (31.4%, n=11), and fear of perpetrators (22.8%, n=8).

Attitude towards sexual violence

**Figure 5:**
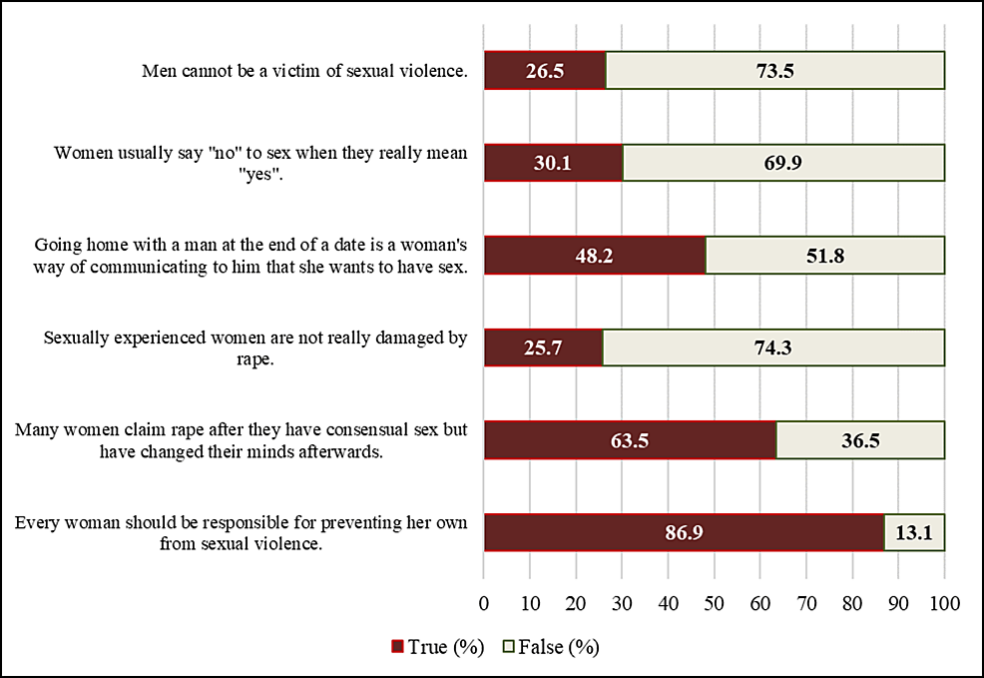
Attitude towards sexual violence among female students of DBU, May 2016 DBU: Debre Berhan University

The majority of the participants (35.7%, n=224) said that they would confide in their health professionals, 28.5% (n=179) said they would report to the police or legal authorities, 20.1% (n=126) to a friend, 19.8% (n=124) to a family member, and 10% (n=63) said that they would not tell anyone (Figure 5).

Predictors of sexual violence

Table 3 depicts the predictors of sexual violence among female students in DBU. On bivariate analysis, rape was significantly associated with substance (drug and Khat) use, cigarette smoking, parents’ educational status, year of study, and relationship status. However, after controlling for all covariates, the multivariate logistic regression result showed that only relationship status, study year, and chewing Khat were significantly associated with rape. Students who reported to be in a relationship but not married, currently married, and previously married were more likely to have been raped than students who reported to being single: adjusted odds ratio (AOR)=5.21 (95% CI=1.51-18.04), AOR=27.03 (95% CI=3.96-184.5), AOR=131.9 (95% CI=3.36-5170) respectively (Table 3). Students who were in years two and four were less likely to report rape than year-one students: AOR=0.08 (95% CI=0.01-0.52) and AOR=0.05 (95% CI=0.003-0.87) respectively. Students who reported having a habit of chewing Khat were found to be 16.7 times more at risk of being a rape victim: AOR=16.7 (95% CI=1.24-227.3).

**Table 3:** Factors associated with female students’ sexual violence experience: binary logistic regression analysis, May 2016 *At least one of the three types of sexual violence; **p-value less than 0.05; ***use of cocaine, shisha, marijuana, etc. COR: crude odds ratio; AOR: adjusted odds ratio (adjusted for all covariates); CI: confidence interval

Variable	Rape		Attempted rape		Sexual harassment		Any of the sexual violence acts*	
	COR (95% CI)	AOR (95% CI)	COR (95% CI)	AOR (95% CI)	COR (95% CI)	AOR (95% CI)	COR (95% CI)	AOR (95% CI)
Age in years								
18-20	1	1	1		1	1	1	1
21-29	1.50 (0.88-2.56)	1.53 (0.38-6.11)	1.10 (0.69-1.76)		1.38 (1.01-1.89)**	1.80 (0.91-3.58)	1.39 (1.02-1.91)	1.72 (0.86-3.42)
Relationship status								
Single	1	1	1	1	1	1	1	1
In a relationship but not married	3.55 (1.96-6.42)	5.21 (1.51-18.04)	1.88 (1.16-3.04)	2.54 (1.06-6.01)	1.83 (1.30-2.58)	1.67 (0.92-3.04)	2.18 (1.54-3.09)	1.84 (1.004-3.38)
Married	10.3 (3.90-27.4)	27.03 (3.96-184.5)	0.82 (0.18-3.65)	1.03 (0.14-7.83)	1.05 (0.45-2.44)	0.65 (0.18-2.34)	1.69 (0.72-4.02)	1.11 (0.31-3.98)
Previously married	6.46 (0.64-65.0)	131.9 (3.36-5170)	0.000	0.000	3.45 (0.35-33.4)	4.00 (0.25-62.25)	3.27 (0.34-31.7)	2.38 (0.16-33.8)
Year of study								
One	1	1	1	1	1	1	1	1
Two	0.82 (0.40-1.68)	0.080 (0.01-0.52)	0.68 (0.36-1.27)	0.69 (0.22-2.25)	0.63 (0.42-0.94)	0.28 (0.13-0.61)	0.71 (0.47-1.06)	0.31 (0.15-0.66)
Three	1.68 (0.82-3.46)	0.48 (0.10-2.33)	1.48 (0.79-2.79)	2.35 (0.66-8.30)	1.32 (0.82-2.14)	0.71 (0.29-1.71)	1.34 (0.82-2.16)	0.68 (0.28-1.65)
Four	0.76 (3.46-2.37)	0.05 (0.003-0.87)	0.77 (0.29-1.98)	0.28 (0.03-3.01)	0.845 (0.46-1.55)	0.33 (0.11-1.01)	0.98 (0.54-1.82)	0.38 (0.13-1.19)
Five	0.91 (0.36-2.29)	0.31 (0.03-2.86)	0.59 (0.24-1.42)	0.64 (0.11-3.89)	1.14 (0.67-1.94)	0.44 (0.14-1.45)	1.18 (0.69-2.02)	0.50 (0.15-1.65)
Place of childhood residence								
Rural area	1	1	1	1	1		1	
Urban area	0.68 (0.39-1.15)	1.18 (0.25-5.48)	0.69 (0.43-1.11)	1.07 (0.34-3.35)	0.97 (0.71-1.33)		0.97 (0.70-1.33)	
Current living condition								
On campus using university cafe	1	1	1	1	1	1	1	1
On campus but none-cafe	0.61 (0.36-1.05)	0.35 (0.09-1.24)	0.48 (0.30-0.79)	0.42 (0.15-1.09)	1.35 (0.98-1.85)	1.21 (0.63-2.32)	1.31 (0.95-1.80)	1.05 (0.55-2.02)
Outside the campus	1.21 (0.14-10.3)	0.60 (0.01-53.02)	0.80 (0.09-6.81)	0.000	1.47 (0.32-6.67)	0.93 (0.09-9.42)	2.41 (0.46-12.6)	2.61 (0.19-35.37)
Father’s educational status								
Illiterate	1	1	1	1	1	1	1	1
Primary school	0.45 (0.22-0.93)	0.46 (0.12-1.78)	1.18 (0.67-2.10)	1.47 (0.51-4.33)	1.10 (0.73-1.66)	0.68 (0.31-1.55)	0.97 (0.64-1.48)	0.69 (0.31-1.56)
High school	0.53 (0.93-1.19)	0.38 (0.05-3.25)	0.82 (0.40-1.68)	1.49 (0.33-6.74)	0.76 (0.47-1.23)	0.42 (0.14-1.25)	0.69 (0.43-1.12)	0.44 (0.15-1.31)
College graduate	0.49 (0.25-1.00)	0.66 (0.08-5.04)	0.49 (0.24-1.00)	0.54 (0.09-3.22)	1.28 (0.85-1.95)	1.05 (0.340-3.25)	1.26 (0.83-1.92)	1.18 (0.37-3.70)
Mother’s educational status								
Illiterate	1	1	1	1	1		1	
Primary school	0.57 (0.29-1.15)	0.88 (0.24-3.21)	1.22 (0.71-2.11)	1.12 (0.39-3.14)	0.94 (0.62-1.40)		0.85 (0.56-1.26)	
High school	0.63 (0.30-1.33)	0.23 (0.02-2.35)	0.43 (0.19-0.96)	0.08 (0.01-0.88)	0.86 (0.55-1.34)		0.82 (0.53-1.27)	
College graduate	0.34 (0.13-0.90)	0.54 (0.05-5.96)	0.497 (0.22-1.10)	0.53 (0.08-3.39)	1.15 (0.73-1.83)		1.16 (0.73-1.85)	
Parents’ monthly income								
≤3,000 Birr	1		1		1	1	1	1
>3,000 Birr	0.80 (0.37-1.73)		0.84 (0.43-1.65)		1.42 (0.89-2.26)	1.24 (0.63-2.43)	1.43 (0.89-2.28)	1.30 (0.66-2.55)
Cumulative GPA								
≤2.80	1	1	1	1	1		1	
>2.80	0.68 (0.37-1.25)	0.63 (0.21-1.91)	0.64 (0.38-1.06)	0.41 (0.17-1.00)	1.07 (0.76-1.52)		0.98 (0.69-1.38)	
Cigarette smoking								
Never	1		1	1	1	1	1	1
Smokes cigarette	4.34 (1.29-14.5)	1.33 (0.03-58.63)	4.49 (1.43-14.08)	0.57 (0.01-31.4)	11.54 (1.49-89.3)	2.65 (0.000)	10.19 (1.32-78.9)	3.21 (0.000)
Alcohol consumption								
Never	1	1	1	1	1	1	1	1
Drink alcohol	1.62 (0.87-2.97)	1.21 (0.31-4.71)	1.78 (1.04-3.05)	1.67 (0.60-4.66)	2.88 (1.86-4.47)	1.48 (0.68-3.22)	2.75 (1.76-4.29)	1.38 (0.64-2.99)
Chewing Khat								
Never	1	1	1	1	1		1	
Chew Khat	9.04 (3.16-25.8)	16.7 (1.24-227.3)	2.56 (0.79-8.25)	3.63 (0.17-76.11)	1.574 (0.000)		0.99 (1.390E9-0)	
Drug use***								
Never	1	1	1		1	1	1	1
Use drugs	5.53 (1.79-17.0)	1.80 (0.10-32.38)	1.89 (0.51-6.95)		3.49 (0.96-12.6)	0.18 (0.01-2.71)	3.08 (0.85-11.16)	0.17 (0.01-2.52)

Being a victim of attempted rape was significantly associated with relationship status, current living conditions, mother’s educational status, cigarette smoking, and alcohol drinking in bivariate analysis. However, only the mother’s educational status was found to be significantly associated with attempted rape after the multivariate analysis. Students who reported having a mom with a high school educational level were 92% less likely to have been a victim of attempted rape than students with illiterate moms (AOR=0.08; 95% CI=0.01-0.88).

Likewise, sexual harassment was significantly associated with age, relationship status, year of study, cigarette smoking, and alcohol consumption in the bivariate analysis; however, after controlling for all covariates, only the year of study was found to be significantly associated. Year-two students were 72% less likely to report sexual harassment than year-one students (AOR=0.28; 95% CI=0.13-0.61).

Lastly, the occurrence of any of the three sexual violence types was significantly associated with age, relationship status, cigarette smoking, and alcohol consumption in the bivariate analysis. However, multivariate analysis results showed that relationship status and year of study were the only covariates that were significantly associated with overall sexual violence. Students who were in non-marital relationships were 84% (AOR=1.84; 95% CI=1.004-3.38) more at risk of any of the three sexual violence types than single students. In addition, year-two students were 70% (AOR=0.31; 95% CI=0.15-0.66) less likely to encounter any of the three sexual violence types.

## Discussion

Out of the 627 participants, 61 (9.8%) and 10 (1.6%) had been victims of completed rape in their life and in DBU respectively. Sixteen (26.2%) of the victims had been victims of completed rape more than once. This study finding is almost similar to a study conducted in Chile on the prevalence of and risk factors for sexual victimization, which revealed that 9% of rape was reported among college students [27]. A study in Addis Ababa has also revealed a close result: that the prevalence of completed rape and attempted rape was 5% and 10%, respectively [11]. On the contrary, a study conducted among female students of Haramaya University reported much lower rates than this one, which was 3% lifetime rape and 19.3% sexual harassment [12]. This could be partly because the authors used only one parameter to measure sexual harassment, which is "encountered unwelcome touch on private bodies".

The findings of this study are comparable with those of a cross-sectional survey conducted among female students at the Addis Ababa University in 2004, in which the prevalence of lifetime completed and attempted rape was 12.7% and 27.5%, respectively, whereas sexual harassment reported in a lifetime and 12-month period was 58% and 41.8%, respectively [11].

The rape report rate that this study found in DBU (1.6%) is almost similar to a study in South Africa, which found that in the course of a year, 1.3% of women (aged 18-49 years) had been forced, physically or by means of verbal threats, to have non-consensual sex [12]. The finding of this study is very low compared to a 2013 study in Hawassa University, which found instances of 14.3% of completed rape among students since being admitted to the university and 3% in just the past year [28]. This could be due to reporting bias in the previous study. The finding is also similar to the finding of another study conducted among female college students in Mekelle, which reported that 34.4% had experienced sexual violence since joining college [29].

Sexual harassment was found to be the most common form of sexual violence ever experienced by female students (51.8%). More than one in three female students (34.4%) have been a victim of sexual harassment while they are in DBU, which is lower than reported in a 2004 study at Addis Ababa University, which documented a sexual harassment rate of 74.3% [11]. The finding of lifetime sexual harassment was almost similar to the study in Addis Ababa, in which 58% of students reported experiencing sexual harassment in their lifetime [11].

In a study conducted at Addis Ababa University, female students reported a similar finding in rape prevalence, 12.7% [11]. However, the same study reported a lifetime attempted rape rate of 27.5%, which is very high compared to the current study. This could be due to the small sample size in the previous study. The same study reported that 58% of female students had experienced at least one form of sexual harassment in their lifetime, which is comparable to our study. This study finding was higher than a report from Haramaya University, which revealed that 3% of females experienced rape in their lifetime [12]. This could be explained by the remoteness of the latter university, which is located far from any urban area.

In this study, among the factors significantly associated with rape, relationship status, study year, and chewing Khat retained their significance even after controlling for other covariates. Multiple logistic regression analyses revealed that Khat-chewing habit and marital status were significantly associated with rape.

Mothers' educational status was the only factor significantly associated with attempted rape. Students who reported having a mom with a high school education were less likely to have been a victim of attempted rape than students with illiterate moms. This could be explained by the fact that educated moms may teach their daughters about sexual violence, which might help them protect themselves better.

Year-two students were less likely to be rape and sexual harassment victims than year-one students and were less likely to experience any of the three sexual violence types compared to year-one students. Similarly, students who were in year four were less likely to be rape victims than year-one students.

Although studies have reported that sexual violence cases often involve alcohol consumption by the perpetrator, victim, or both [30], in our study, alcohol consumption was not associated with any of the sexual violence types when controlled for other covariates. Students who were in a non-marital relationship were at a higher risk of experiencing either of the three sexual violence types than single students, even though it is not clear if being in a non-marital relationship is a cause or consequence.

This study has some limitations, including possible under-reporting of the outcome variables (especially rape) because of the sensitivity attached to the subject of sexual violence. In addition, the study could most likely have been affected by recall bias since it deals with past experiences. Hence, the findings of the study should be interpreted with caution.

## Conclusions

The study revealed that the prevalence of sexual violence, in general, was high among DBU female students, and sexual harassment was the most common form of sexual violence ever experienced by female students followed by attempted rape and rape. Hence, parents and legal guardians of girls should be aware of the risk of sexual violence by individuals who are close to the girls and take caution to protect the girls. The university should also raise awareness among female students about sexual violence and urge them to report sexual violence; the university authorities should encourage survivors of sexual violence to seek medical care and provide information as to where to go for treatment and legal advice if they experience sexual violence of any sort. Further broad and longitudinal studies are needed to determine the predictors of the problem among female students in DBU and Ethiopia as a whole.
